# Psychological resilience, athletic experience, and competitive level of judokas. A transversal study

**DOI:** 10.3389/fpsyg.2024.1440412

**Published:** 2024-07-31

**Authors:** María Garrido-Muñoz, Cecilia Blanco-García, Ignacio Diez-Vega, Sonia García-Merino, Jorge Acebes-Sánchez, Gabriel Rodríguez-Romo

**Affiliations:** ^1^Deporte y Entrenamiento Research Group, Departamento de Deportes, Faculty of Physical Activity and Sport Sciences (INEF), Universidad Politécnica de Madrid, Madrid, Spain; ^2^Departamento de Enfermería y Fisioterapia, Facultad de Ciencias de la Salud, Universidad de León, León, Spain; ^3^Faculty of Psychology, Universidad Francisco de Vitoria (UFV), Madrid, Spain; ^4^Faculty of Health Sciences, Universidad Francisco de Vitoria (UFV), Madrid, Spain; ^5^Centro de Investigación Biomédica en Red de Fragilidad y Envejecimiento Saludable (CIBERFES), Instituto de Salud Carlos III, Madrid, Spain

**Keywords:** judo, sports, psychological resilience, sports performance, CD-RISC 10

## Abstract

**Introduction:**

While there is agreement on the positive link between psychological resilience and athletic performance, conclusive findings regarding the association between psychological resilience and other variables of interest (for example, age, gender, type of sport, or competitive level) remain elusive.

**Objective:**

The study aimed to assess psychological resilience levels among judokas and explore potential associations with demographic factors, judo experience and competitive level.

**Methods:**

A total of 702 judokas (469 men and 233 women) participated in the study, of whom 194 (27.6%) were classified as TOP by their competitive level. Psychological resilience was evaluated using the Spanish version of the *10 item Connor-Davidson Resilience Scale* (CD-RISC 10), with a score range from 0 to 40. Independent *T*-test and Pearson’s coefficient were used for bivariate analysis. A two-way non-parametric ANCOVA was carried out to analyse the impact of gender and competitive level on psychological resilience.

**Results:**

The judokas showed total mean scores in the CD-RISC 10 of 33.08 points (SD = 4.79), considered high. Levels of psychological resilience were significantly higher among men (33.36 ± 4.76) than women (32.53 ± 4.80) and were positively correlated with age and number of years practicing and competing in Judo (*p* = 0.019). Judokas with a higher competitive level (TOP judokas) showed significantly higher levels of resilience than the others (non-TOP judokas) (*p* < 0.001). These differences in resilience according to competitive level persisted, among both men and women, when adjusting the model of analysis (two-way ANCOVA) for all variables considered in the study, although with a small effect size.

**Conclusion:**

The results suggest that the practice of Judo, especially over long periods of time, is associated with high scores in psychological resilience. Furthermore, psychological resilience appears to be a differentiating variable among judokas at a high-competitive level, and its evaluation and development using different strategies based on age and gender should be considered by trainers and psychologists.

## 1 Introduction

Judo is a martial art of Japanese origin, created in 1882 by Jigoro Kano. Over the course of the 20th century, and in the wake of the Second World War, Judo was gradually transformed into a competitive sport, quickly becoming extremely popular throughout the world. Currently, Judo is practiced is over 200 countries on five continents (Brousse and Messner, n.d.). The development of Judo from a martial art to an Olympic sport, was logically accompanied by efforts to maximize the performance of competitive judokas. In this line, Piskorska et al. (2016) note that the psychological characteristics and personality traits of athletes, and their cognitive abilities, are particularly important in terms of performance in combat sports. Certainly, all competitive sports demand maximum effort by athletes, involving acute mental stress and physical fatigue. However, combat sports involve a series of specific circumstances and characteristics which may explain the possibly greater salience of psychological and personality factors in terms of athletic performance, such as direct physical contact and attack against an opponent, the habitual weight loss before competitions, the risk of pain and possible injury, as well as the persistent fear of defeat.

Within this context, psychological resilience has been identified as an important factor in athletic performance, either in general terms or in specific types of sports (Galli and Vealey, 2008; Hosseini and Besharat, 2010; Fletcher and Sarkar, 2012; Belem et al., 2014; Meggs et al., 2016), including combat sports (Litwic-Kaminska, 2013; Yang et al., 2019). Psychological resilience has been defined as both a trait and a process. As a trait, it refers to a set of fixed and stable personal characteristics that allows an individual to adapt or protect themselves from diverse sources of significant levels of stress or trauma, enabling them to cope (Connor and Davidson, 2003; Lee et al., 2013). Other authors consider psychological resilience to be a process, that is, a dynamic response which enables positive adaptation to adversity (Luthar et al., 2003). In this case, the influence of personal characteristics is not fixed but varies according to the situation, the personal circumstances of the moment and the intensity of risk factors (Rutter, 2006). Regardless of whether resilience is a process or a trait, these definitions underscore the idea that psychological resilience implies the ability to face and to adapt effectively to adversity, stress, or traumatic events and to emotionally recover. Additionally, a systematic review in sport psychology introduced the concept of “Sporting Resilience,” emphasizing a resilience filter composed of biopsychosocial protective factors, which influence the impact of adversity and the trajectory of positive adaptation (Gupta and McCarthy, 2022). During the COVID-19 lockdowns, athletes faced significant constraints in training, leading to physical and mental hardship. Research indicated that decreases in training volume, lower lockdown-specific resilience, and negative perceptions about mobility restrictions contributed to perceived barriers to training, underscoring the importance of resilience in overcoming such challenges (Harman et al., 2022). Additionally, a qualitative study highlighted the challenges faced, support systems utilized, and the role of well-being practices like mindfulness and self-care in fostering resilience, promoting long-term psychological well-being (Hussain et al., 2023).

In line with the broad consensus on the association between greater psychological resilience and optimum athletic performance, several researchers have specifically analyzed whether athletes at higher competitive levels show higher levels of psychological resilience (Castro-Sánchez et al., 2016; Blanco-García et al., 2021). However, both studies found no association between these variables. Given these results, Blanco-García et al. (2021) suggested that optimum athletic performance does not necessarily correspond to athletes at higher competitive levels (high level athletes) but rather athletes, regardless of their category or level, make effective and efficient use of their capacities and resources to achieve the best sports results possible. Thus, the positive relation between psychological resilience and athletic performance found in previous studies (Hosseini and Besharat, 2010; Litwic-Kaminska, 2013; Belem et al., 2014; Yang et al., 2019) may be found regardless of the competitive level of athletes.

A few studies have attempted to determine if psychological resilience is an equally important variable across different sports. In this line, studies by Chacón-Cuberos et al. (2016) and Boghrabadi et al. (2015) found no significant differences in the levels of resilience among athletes of different types of individual and team sports. However, divergent results have been found in evaluations comparing the psychological resilience of athletes of combat sports and those of other sports (individual and team sports). For example, Reche-García et al. (2020) concluded that athletes of combat sports have significantly higher levels of psychological resilience compared to those practicing individual or team sports. By contrast, Bingol and Bayansalduz (2016) found no differences in levels of resilience between those practicing team sports (volleyball, basketball, handball, and football) and combat sports (boxing, wrestling, Muay Thai and Taekwondo). Similarly, Blanco-García et al. (2021) in their study of a large sample of competitive athletes found no relation between resilience and the type of sport practiced (basketball, handball, volleyball, athletics, and Judo).

Age and gender are the most analyzed variables in studies into psychological resilience in sports. Regarding age, some researchers have found a direct and positive association between age and levels of psychological resilience in specific samples of athletes (Codonhato et al., 2018a; Blanco-García et al., 2021). By contrast, other studies also conducted with athletes found no relation between these variables (Chacón-Cuberos et al., 2016; Tutte and Reche-García, 2016; Reche-García et al., 2020). In terms of gender, various authors found significantly higher levels of resilience among male than female athletes (Zurita-Ortega et al., 2017; Biricik and Sivrikaya, 2020; Blanco-García et al., 2021). However, other studies found no relation between gender and psychological resilience (Hosseini and Besharat, 2010; Boghrabadi et al., 2015; Bingol and Bayansalduz, 2016). For young female athletes, who face distinct challenges such as sport inequity, body image issues, and increased mental distress, fostering resilience is particularly important for enhancing performance and personal growth (O’Brien et al., 2021).

In summary, it may be said that psychological factors have a significant influence on performance in combat sport, especially during competition matches (Piskorska et al., 2016; Rossi et al., 2022). Furthermore, there is broad consensus on the positive relation between psychological resilience and athletic performance in sports (Galli and Vealey, 2008; Hosseini and Besharat, 2010; Fletcher and Sarkar, 2012; Litwic-Kaminska, 2013; Belem et al., 2014; Meggs et al., 2016; Yang et al., 2019). Nevertheless, despite numerous studies, no conclusive evidence has been found of possible associations between psychological resilience and other variables of interest such as, gender, age, type of sport or competitive level. Thus, several authors have noted the need for further research to identify these associations by using larger and more heterogenous samples (Codonhato et al., 2018a,b; Bicalho et al., 2020), with the aim of overcoming the limitations of previous studies.

Considering the above, the following research questions were raised: could resilience be a prominent psychological quality among judokas? What role do variables such as gender, age and experience (practicing and competing) of judokas play at their levels of psychological resilience? Is psychological resilience a differential variable among high-level competitive judokas?

To try to answer these questions, the aim of this study was to evaluate the levels of psychological resilience of a large sample of judokas, and their possible associations with demographic variables, variables related to level of experience in practicing Judo and variables related to competitive level. Furthermore, possible differences in resilience were specifically evaluated with reference to the competitive level in Judo and gender.

In relation to these objectives, the following hypotheses were proposed: (i) judokas will present high levels of psychological resilience; (ii) levels of psychological resilience will be significantly higher in male judokas than in female judokas; (iii) the levels of psychological resilience will be directly and significantly correlated with age, as well as with the variables related to the experience and the competitive level of the judokas; and (iv) the judokas with a higher competitive level will present higher psychological resilience values than the rest of judokas, both in men and women.

## 2 Materials and methods

### 2.1 Design and participants

A descriptive and transversal study was conducted using an on-line questionnaire designed specifically for the project. The target population was active judokas at least 18 years of age and residents of Spain.

The sample was selected using a nonprobability and incidental snowball sampling method (Browne, 2005) based on the personal contacts of researchers, the social media of region Judo federations and the principal Judo clubs in Spain. A total of 726 surveys were collected. After filtering the responses, the final sample consisted of 702 active judokas.

Participation in the study was voluntary and entirely confidential. The survey includes an introductory page which outlined the origins and objectives of the study along with ethics information and required the express consent of participants before beginning the survey. Ethical approval for this study was obtained from the Ethics Research Committee of Universidad Politécnica de Madrid (Ref. number FDRED00000-DML-DATOS-20230609).

### 2.2 Instrument and variables

Four groups of variables were analyzed: demographic (age and gender), psychological (resilience), variables related to experience in the practice of Judo (starting age in practicing Judo; years practicing Judo; hours of practice per week and years competing where applicable) and variables related to competitive level: national team [to be a current or former member of the national junior and/or senior Judo team (yes or no)] and high-level athletes (HLA) or high-performance athletes (HPA) [to be or have been officially recognized by the Spanish Sports Council as a HLA and/or HPA (yes or no)].

An additional variable was created to further classify Judokas into two groups according to their competitive level: TOP (including participants who responded, “yes” to the variables “national team” and/or “HLA or HPA”) and non-TOP (not meeting any of these two criteria).

The *10-item Connor-Davidson Resilience Scale* (CD-RISC 10) (Campbell-Sills and Stein, 2007) was used to evaluate psychological resilience. This is a 10-item, self-report scale of developed to address certain limitations found in the factor structure of the original 25-item *Connor-Davidson Resilience Scale* (CD-RISC) (Connor and Davidson, 2003).

The instrument uses a five-point Likert type scale (0 = never; 4 = almost always). The final scores of the questionnaire are a total of the responses for each item with a total possible score of 40. Higher scores indicate higher levels of resilience.

The CD-RISC 10 has proven to be a stable scale with excellent psychometric properties (Campbell-Sills and Stein, 2007). The reliability and validity of the Spanish version of the CD-RISC 10 was evaluated by Notario-Pacheco et al. (2011) among young adults showing good psychometric properties, a high degree of reliability (Cronbach’s *α* = 0.85) and corroborating its single factor structure. For the present study, the reliability of the CD-RISC 10 was also high, with very similar results (Cronbach’s *α* = 0.87).

### 2.3 Procedure

The final version of the survey was formatted using Google Forms, including questions on the variables of the study and the 10-item CD-RISC. The survey was loaded into the online Google surveys platform. Using the personal contacts of the researchers, the survey was distributed through WhatsApp groups, email, and social media (Facebook, Twitter, and official websites) to the principal regional federations and Judo clubs in Spain using the snowball sampling method (Browne, 2005).

The questionnaire was made available online for 3 months (from October 1, 2020, to December 31, 2020). Responses were anonymous and the participants had an unlimited time to compete the survey, although the maximum time required was approximately 10 min. Upon conclusion of the survey admissions period, these were reviewed and filtered, eliminating contradictory responses and empty or incomplete surveys.

### 2.4 Statistical analysis

The data collected in the questionnaires were analyzed using the Statistical Package for the Social Sciences (SPSS v29) (IBM, Armonk, NY, United States). A descriptive analysis was performed to explore the sample characteristics.

The sample characteristics are described by frequency, percentage, mean (*M*) and standard deviation (SD). Before running inferential tests, parametric assumptions were checked.

Independent *T*-test and Pearson’s coefficient were used to compare the differences [gender, HLA/HPA (yes/no), national team (yes/no) and competitive level (TOP/non-TOP)] or study the association (age, starting age in practicing judo, years practicing judo, hours of practice per week and years competing in judo) based on the resilience scores of the sample. In addition, the Independent *T*-test was used again to compare the characteristics of the participants (age, starting age in practicing judo, years practicing judo, hours of practice per week and years competing in judo) based on their competitive level. A Pearson chi square test was used to compare gender distribution according to judokas competitive level.

A two-way ANCOVA was carried out to analyze the impact of gender and competitive level on psychological resilience (CD-RISC 10 total score), adjusting by the main quantitative variables analyzed (age, starting age in practicing Judo, years practicing Judo, hours of practice per week and years competing in Judo). Due to the violation of the normality assumption in some variables, a Quade’s non-parametric ANCOVA procedure was performed to improve the robustness of the estimates.

Significant level was set at *p* = 0.05 and partial eta squared (*η*^2^*_p_*) was used as effect size estimator.

## 3 Results

The sample consisted of 702 practitioners of Judo, of whom 469 (66.8%) were men (age, *M* = 40.32; SD = 14.11 years) and 233 (33.2%) were women (age, *M* = 31.46; SD = 10.97 years). By their competitive level, 194 Judokas (27.6%) were classified as TOP. Table 1 shows the principal characteristics and distribution of the sample.

**Table 1:** Sample distribution.

Variables	Total	Male	Female
Age^a^	37.38 (13.79)	40.32 (14.11)	31.46 (10.97)
Starting age in Judo’s practice^a^	11.29 (10.06)	11.77 (10.32)	10.32 (9.47)
Years practicing Judo^a^	22.84 (14.16)	24.42 (14.95)	19.65 (11.81)
Hours of practice per week^a^	8.13 (7.24)	7.71 (7.28)	8.95 (7.09)
Years competing in Judo^a^	8.87 (9.44)	8.57 (9.90)	9.47 (8.40)
**HLA/HPA**^b^			
No	543 (77.4)	404 (86.1)	139 (59.7)
Yes	159 (22.6)	65 (13.9)	94 (40.3)
**National team**^b^			
No	570 (81.2)	422 (90.0)	148 (63.5)
Yes	132 (18.8)	47 (10.0)	85 (36.5)
**Competitive level**^b^			
Non-TOP	508 (72.4)	380 (81.0)	128 (54.9)
TOP	194 (27.6)	89 (19.0)	105 (45.1)
Total^b^	702 (100)	469 (66.8)	233 (33.2)

Results are expressed in: ^a^ mean (standard deviation); ^b^ frequency (percentage).

HLA/HPA, high-level athletes/high-performance athletes.

Table 2 shows the associations between the different analyzed variables for the sample of judokas and the total scores obtained in the CD-RISC 10. Regarding demographic variables, significant differences were found in terms of gender (*p* = 0.030), with men scoring higher (*M* = 33.36; SD = 4.76) than women (*M* = 32.53; SD = 4.80) in psychological resilience. A significant and positive association (*p* = 0.035) was also found between age and total scores in the CD-RISC 10.

**Table 2:** Associations of CD-RISC 10 total score with the scale and categorical variables analyzed.

	CD-RISC 10 total score			
Variables	Mean	SD	*r/t*	*p*
Age	–	–	0.080	**0.035**
Starting age in practicing Judo	–	–	0.017	0.647
Years practicing Judo	–	–	0.089	**0.019**
Hours of practice per week	–	–	0.057	0.130
Years competing in Judo	–	–	0.161	**<0.001**
**Gender**				
Male	33.36	4.76	2.175	**0.030**
Female	32.53	4.80		
**HLA/HPA**				
No	32.77	4.88	−3.473	**<0.001**
Yes	34.16	4.29		
**National team**				
No	32.78	4.89	−4.001	**<0.001**
Yes	34.41	4.05		
**Competitive level**				
Non-TOP	32.64	4.94	−4.374	**<0.001**
TOP	34.25	4.14		
Total	33.08	4.79	–	–

SD, standard deviation; *r*, Pearson’s correlation; *t*, *t*-value; *p*, *p*-value.

HLA/HPA, high-level athletes/high-performance athletes.

Significant values are shown in bold.

Regarding the variables related to experience in practicing Judo, the results show a significant positive association between total scores in the CD-RISC 10 and the number of years practicing (*p* = 0.019) and competing in Judo (*p* < 0.001). However, no significant association was found with the starting age in practicing Judo (*p* = 0.647), nor the number of hours of practice per week (*p* = 0.130).

Finally, significant differences in levels of psychological resilience were found according to variables related to the competitive level of Judokas: national Judo team, HLA/HPA and TOP/non-TOP (*p* < 0.001, in all cases). Scores for resilience were significantly higher among judokas who are or were members of the national junior and/or senior Judo team (*M* = 34.41; SD = 4.05) than among those who were not (*M* = 32.78; SD = 4.89). Judokas who are or were officially recognized as HLA and/or HPA also obtained higher scores in resilience (*M* = 34.16; SD = 4.29) than those who were not (*M* = 32.77; SD = 4.88). Finally, judokas included in the TOP category, based on their competitive level, obtained higher scores (*M* = 34.25; SD = 4.14) than judokas non-TOP (*M* = 32.64; SD = 4.94) (see Table 2).

The classification of the sample of judokas by their competitive level (TOP vs. non-TOP) revealed significant differences in terms of gender [χ^2^(1) = 52.976; *p* < 0.001], with a higher percentage of women TOP (45.1%; *n* = 105) than men (19.0%; *n* = 89). Additionally, as shown in Table 3, there were also significant differences (*p* < 0.001 in all cases) among TOP and non-TOP judokas in the rest of the variables (starting age in practicing Judo, years practicing Judo, hours of practice per week and years competing in Judo), except for age (*p* = 0.272).

**Table 3:** Sample distribution data according to competitive level (TOP vs. non-TOP).

	Competitive level			
Variables	TOP	Non-TOP	*t*	*p*
Age	36.45 (14.35)	37.73 (13.57)	1.099	0.272
Starting age in practicing Judo	7.15 (4.19)	12.87 (11.14)	9.880	**<0.001**
Years practicing Judo	27.79 (13.03)	20.95 (14.12)	−5.864	**<0.001**
Hours of practice per week	11.97 (8.73)	6.66 (5.97)	−7.802	**<0.001**
Years competing in Judo	15.07 (7.60)	6.50 (8.99)	−12.669	**<0.001**

Results are expressed in mean (standard deviation); *t*, *t*-value; *p*, *p*-value.

Significant values are shown in bold.

**Figure 1:**
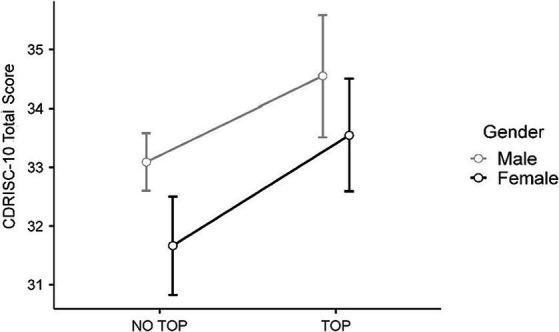
CD-RISC 10 total score by competitive level and gender.

Considering the associations observed between certain variables and psychological resilience, and differences found in the distribution of the sample according to competitive level, a two-way a Quade’s non-parametric ANCOVA procedure was conducted to evaluate the possible association between competitive level and gender with psychological resilience (total score in the CD-RISC 10), and the possible interaction between these variables. The model was adjusted to evaluate all the quantitative variables analyzed (age, starting age in practicing Judo, years practicing Judo, hours of practice per week and years competing in Judo). The results show significant differences in scores for resilience according to gender [*F* (1, 698) = 8.539, *p* = 0.004, *η*^2^*_p_* = 0.012], with higher adjusted mean scores among men (*M* = 33.82; SE = 0.28) than women (*M* = 32.61; SE = 0.32). Significant differences were also found in total scores in the CD-RISC 10 according to the competitive level of judokas [*F* (1, 698) = 8.233, *p* < 0.004, *η*^2^*_p_* = 0.012], with higher mean adjusted scores among TOP judokas (*M* = 34.05; SE = 0.37) than non-TOP (*M* = 32.38; SE = 0.25). However, no significant association was found between gender and competitive level of athletes and their scores for psychological resilience [*F* (1, 698) = 0.007, *p* = 0.932] (see Figure 1).

## 4 Discussion

The present study had a twofold objective. First, to evaluate the levels of psychological resilience of a large sample of judokas, and their possible associations with demographic variables, variables related to level of experience in practicing Judo and variables related to competitive level. Secondly, possible differences in psychological resilience were specifically evaluated with reference to the competitive level in Judo and gender.

Regarding levels of psychological resilience, the sample of judokas participating in the study had a mean total score of 33.08 (SD = 4.79) in the CD-RISC 10. This score may be considered high according to the score ranges used to interpret the scale (Davidson, 2018). There are factors, such as the characteristics and distribution of the sample, which limit any direct comparison of these scores with the results of other studies which also used the CD-RISC 10 to evaluate resilience either among the general healthy adult population or among athletes. This considered, the judokas participating in this study scored significantly higher in psychological resilience than the participants in other previous studies, which allows confirming the first hypothesis that was raised in the present study.

Davidson (2018) notes that in two surveys conducted on the general healthy adult population in the United States, mean scores in the CD-RISC 10 were between 31 and 32 points. However, some studies conducted in other countries show substantially lower mean scores, suggesting that ethno-cultural factors must be considered in measuring psychological resilience (Davidson, 2018). In this case, for example, studies on the general healthy adult population in Brazil (Lopes et al., 2011), Greece (Kyriazos et al., 2021), and Spain (Antúnez et al., 2015), showed mean scores between 28 and 29 points.

Specifically in the field of sports, research into adult competitors in other sports showed mean scores in the CD-RISC 10 ranging from 29 to 31 points (Gucciardi et al., 2011; Gonzalez et al., 2016; Belem et al., 2017; Codonhato et al., 2018a,b). Among these, the study with the highest scores (31 points) was that by Belem et al. (2017) using a sample of 50 male athletes of mixed martial arts.

Other studies which made use of instruments other than the CD-RISC 10 to evaluate psychological resilience, found disparate results in comparing the resilience of judokas or other combat sports with other sports modalities. Reche-García et al. (2020) found significantly higher levels of psychological resilience among athletes of combat sports (fencing, karate or taekwondo, among others) compared to those practicing individual (athletics) or team sports (football and basketball). However, Blanco-García et al. (2021) found no differences in resilience according to the type of sport (basketball, handball, volleyball, athletics, and Judo). Similarly, a study by Bingol and Bayansalduz (2016) found no significant differences in resilience among those practicing team sports (volleyball, basketball, handball, and football) compared to combat sports (boxing, wrestling, Muay Thai and Taekwondo).

The results of the present study indicate an association between the level of psychological resilience of judokas and age and gender. In terms of gender, male judokas scored significantly higher in resilience than female judokas, as had been hypothesized. These differences persisted when the analysis model was adjusted for all the quantitative variables under study, although with a small effect size. These results are in line with the findings of previous studies among healthy adult populations where men also scored higher in resilience than women (Notario-Pacheco et al., 2011; Lee et al., 2013; Cheng et al., 2020; Pulido-Martos et al., 2020; Kavčič et al., 2023). However, other authors note that gender differences in terms of resilience are not conclusive (Lee et al., 2013; Davidson, 2018; Pulido-Martos et al., 2020). While some studies found similar associations as those described, others found no relation between the two (Lopes et al., 2011; Antúnez et al., 2015; Liu et al., 2015).

In the context of sports, the situation is similar. Thus, the results of the present study are in line with the findings of previous studies which also found significantly higher levels of resilience among male than female athletes (Zurita-Ortega et al., 2017; Biricik and Sivrikaya, 2020; Blanco-García et al., 2021). However, other studies into the psychological resilience of athletes found no relation between gender and psychological resilience (Hosseini and Besharat, 2010; Boghrabadi et al., 2015; Bingol and Bayansalduz, 2016).

Regarding the possible relation between age and levels of resilience, the reviewed literature was also far from conclusive. Several studies on the general population reported no differences in resilience according to age (Connor and Davidson, 2003; Pulido-Martos et al., 2020) while others did find a positive association between both variables (Lundman et al., 2007; Portzky et al., 2010; Rodríguez-Rey et al., 2016).

The present study found that the age of judokas is significantly and positively associated with levels of resilience, as had been hypothesized. In the field of sports, these results are in line with those of Codonhato et al. (2018a) and Blanco-García et al. (2021), which also found a positive association between age and resilience among athletes. According to Codonhato et al. (2018b), the relation between age and resilience may be the logical outcome of the concept of resilience itself. Thus, a possible explanation of the positive association between these variables found in some studies may be the diverse range of personal, professional, and social experiences that come with age. It is to be expected that older individuals have more experience in facing personal challenges and adversity, which may lead to greater psychological resilience. However, just as with the studies of the general population, evidence of the association between age and psychological resilience among athletes is also inconclusive. Different research (Chacón-Cuberos et al., 2016; Tutte and Reche-García, 2016; Reche-García et al., 2020), in contrast to the findings of the present study, found no differences in the psychological resilience of athletes according to age.

Various authors have suggested that this disparity in the findings on psychological resilience according to age and gender, among both the general population and specific groups, may be due to methodological reasons. Lee et al. (2013) notes that many studies into these relations used relatively small and homogenous samples. The differences in the type of measurement instrument (and their versions) used to evaluate psychological resilience may also be a factor. Thus, for example, some authors have suggested that the gender differences found in studies using the CD-RISC 10 scale may be due to the fact this scale is focused on specific qualities of resilience which are less prevalent in women than in men (Cheng et al., 2020; Pulido-Martos et al., 2020; Kavčič et al., 2023). Additionally, other factors may result in inconclusive results on the influence of age on resilience, such as the limited age range of the samples using in various studies, along with the use of age as an ordinal variable (classification by age group) which may produce certain biases rather than using the age variable on a continuous scale which may better reflect reality (Pulido-Martos et al., 2020).

Although further research into the influence of age and gender on psychological resilience is necessary, our contribution to this debate is clear. The present study made use of a large sample of judokas, with a broad range of ages (considered on a continuous scale), from 18 to 79 years of age, and with a gender distribution (66.8% men and 33.2% women) which largely reflects that of current federated Spanish judokas (74.1% men and 25.9% women) (CSD, 2023). Using the CD-RISC 10 as an evaluation instrument on the sample of judokas, we find higher levels of psychological resilience among men than women, positively associated with age. Another study on a large and heterogeneous sample of Spanish athletes (Blanco-García et al., 2021) reached the same conclusions despite using a different evaluation instrument (the Spanish version of the Brief Resilience Scale, BRS) (Rodríguez-Rey et al., 2016). Thus, our results point to certain conclusions which should be confirmed by future research.

Regarding the possible relations between levels of psychological resilience and variables related to experience in the practice of Judo, our study found that total scores in the CD-RISC 10 are significantly and positively associated with the number of years practicing and competing in Judo, but not with the starting age in practicing Judo, nor with the number of hours per week practicing. Therefore, our initial hypothesis is only partially confirmed. Similarly, a study by Pujszo et al. (2019) of 76 male athletes of various martial arts (Aikido, Muay Thai/Kickboxing and Judo), concluded that the experience of long-term training (number of years in training) is associated with increased psychological resilience.

As noted in the evaluation of differences in resilience according to age, it is possible that the relation found between number of years practicing and competing in Judo is also logically associated with the concept of psychological resilience itself. If resilience is a process that takes place of over time (Luthar et al., 2003) through lived experience, it is probably that more experienced judokas have faced greater adversity in training and competition, contributing to the acquisition of higher levels of resilience. Similarly, other studies have also found that experience in the practice of sports is associated with greater capacity for emotional adaptation (Rodriguez-Romo et al., 2021) and a greater ability to cope with stress (Laborde et al., 2013). Furthermore, our results are partially in line with those of a study by Reche-García et al. (2020) on a sample of 278 men and women practicing individual, team and combat sports. As in the present study, these authors found no differences in resilience according to number of weekly training sessions. However, contrary to our findings, they found no association between resilience and number of years practicing team or combat sports while among those practicing individual sports resilience was higher among those with fewer years of experience. Here again, methodological factors may explain these results. Reche-García et al. (2020) considers the different variables (number of weekly training sessions, years of practice and resilience) as ordinal variables (classified by groups); the small sample size of some sub-samples of athletes may also explain the disparity in the results.

Finally, the results of the present study show that levels of psychological resilience differ significantly according to all variables related to the competitive level of judokas. Those who are or have been members of the National Judo Team in the junior and/or senior category and those who are or have been officially recognized HLA and/or HPA, show higher levels of resilience than judokas not included in any of these groups. Similarly, judokas included in the TOP category according to their competitive level, scored significantly higher in the CD-RISC 10 than non-TOP judokas. Although the effect size was small, the results show these differences in resilience between TOP and non-TOP judokas persisted regardless of the effect that the rest of the variables analyzed in the present study could have on them or the differences observed in the distribution of the sample based on their competitive level (TOP vs. non-TOP judokas). Further, no interaction effect was found between the variables gender or competitive level, reproducing these differences in both men and women. These results seem to confirm the initially stated hypothesis. Specifically, that judokas with a higher competitive level would present higher values of psychological resilience than the rest of judokas, both in men and women.

These findings are in line with the broad consensus found in existing literature on the positive association between psychological resilience and athletic performance (Galli and Vealey, 2008; Hosseini and Besharat, 2010; Fletcher and Sarkar, 2012; Litwic-Kaminska, 2013; Belem et al., 2014; Meggs et al., 2016; Yang et al., 2019). A study by Hosseini and Besharat (2010), with a sample of different sports athletes, found that resilience is positively associated with athletic achievement and psychological wellbeing, and negatively associated with psychological dysfunction. In the specific case of combat sports, Yang et al. (2019), in their study of taekwondo athletes in secondary school, found that resilience improved the capacity of these athletes to use psychological strengths for enhanced performance. Litwic-Kaminska (2013), in a study of practitioners of Judo and Taekwondo, found that athletes with higher levels of psychological resilience are less likely to regard situations as harmful or in terms of loss. Furthermore, they experienced lower levels of anxiety and habitually employed coping strategies aimed at specific objectives and actions (considered most effective), with a positive impact on their athletic performance.

Nevertheless, some authors note that the positive relation between resilience and performance may be found regardless of the competitive level of athletes and that optimum athletic performance may not necessarily involve being among higher level athletes (Blanco-García et al., 2021). Thus, in contrast to our findings, some studies found no association between psychological resilience and the competitive level of athletes (Castro-Sánchez et al., 2016; Blanco-García et al., 2021). Other, however, such as the study by Patsiaouras (2021) with volleyball players, did find associations between both variables, as in the present study. These authors found that high-level players scored higher than lower-level players in some of the variables associated with psychological resilience. Specifically, a greater ability to concentrate on finding solutions, adopting a healthy lifestyle, and having the capacity to overcome problems and difficulties.

## 5 Limitations and strengths

The present study is not without its limitations. The first is in the cross-sectional and descriptive design of the study itself, which does not permit the identification of causal relationships between the analyzed variables. The selection of the sample was not random, limiting the ability to generalize the results of the study. Furthermore, while the scale used to evaluate resilience (CD-RISC 10) is considered an optimum measurement tool with solid psychometric properties for the evaluation of psychological resilience among athletes (Gucciardi et al., 2011; Gonzalez et al., 2016) it also has certain limitations. Firstly, the scale is based on the conceptualization of psychological resilience as a trait, evaluating the personal resources or qualities which favor positive adaptation to adversity but not necessarily the process of resilience (Gonzalez et al., 2016). Additionally, although the CD-RISC is habitually used in the context of sports, it is a general self-report questionnaire that was not specifically designed for use with athletes. Thus, although it may be useful in identifying the general ability of athletes to overcome adversity, a few authors have criticized its use, and the use of other general evaluation instruments, in measuring psychological resilience in sports considering the factors to be specific to each context (Sarkar and Fletcher, 2013; Sarkar and Fletcher, 2014; Galli et al., 2019). Nevertheless, to date no specific instrument has been developed to evaluate psychological resilience in the context of sport, nor to measure resilience as a process of positive adaptation to adversity (Gonzalez et al., 2016; Galli et al., 2019). For this reason, many researchers continue to use general purpose, self-report instruments in specific contexts.

Despite these limitations, the present study made use of the CD-RISC 10 as a measurement instrument. We believe the use of this instrument is in fact a strength of the study. The CD-RISC 10, along with the original 25-item CD-RISC scale, are among the most widely used quantitative instruments to evaluate psychological resilience, both in the specific area of sports (Bicalho et al., 2020) and among other population groups (Windle et al., 2011; Salisu and Hashim, 2017). This circumstance, considering with the limitations, allows us to compare our results with those of numerous other studies which used the same evaluation instrument. Furthermore, this scale is much shorter (10 items) than others, minimizing response time, avoiding fatiguing participants with a lengthy survey and so avoiding the loss of quality and quantity in the responses. Another strength of this study is the large sample size specifically of judokas, the distribution in terms of gender, largely reflecting the current federated Spanish judokas, and the wide age range of participants. To our knowledge, few quantitative studies on psychological resilience in the field of sports has used a sample of similar characteristics, often characterized by small and homogenous samples (Bicalho et al., 2020) which hinder making comparisons between groups and the generalization of their results.

## 6 Conclusion, practical implications and recommendations for future research

The judokas who participated in this study showed high levels of resilience, which were higher among men than women and positively associated with age. High scores for resilience were also correlated positively to the number of years practicing and competing in Judo. Additionally, judokas at higher competitive levels (judokas TOP) showed higher levels of resilience than other judokas participating in the study (judokas non-TOP). These differences in psychological resilience according to competitive level persist among both men and women regardless of the effect that the rest of the variables studied could have on them.

From a practical point of view, and without intending to establish causality between the analyzed variables, the results of this study suggest that the practice of Judo can be associated with high levels of psychological resilience, especially when practiced over long periods of time. Thus, the practice of Judo may be considered a method to develop these qualities. Psychological resilience appears to be a differentiating variable among judokas at a highly competitive level, and its evaluation and development should be considered by trainers and psychologists in the field of high-performance sports. Differences in levels of resilience according to age and gender also suggest the need to employ different strategies based on these variables to develop the skills and abilities (individual, social and environmental) that will allow athletes to enhance their performance in the face of the adversities and challenges of a competitive environment.

Regarding gender and age differences in levels of psychological resilience, the findings of the present study are clear. However, the inconclusive results on these differences that we found in previous studies, whether with the general population or with the athlete population, point to the need to continue researching along these lines. This same situation is reproduced, in the sports context, when analyzing the possible relationships of psychological resilience with the competitive level, sports experience or the type of sport practiced, with discrepant results in previous literature. A possible explanation for this lack of consensus is that numerous previous studies have been carried out with relatively small and homogeneous samples, making comparisons between groups as well as the possible generalization of their results difficult. Therefore, it would be necessary for future research to be carried out on large and heterogeneous samples, to overcome these limitations.

Likewise, the development of more research, of a longitudinal nature and/or based on specific interventions, is suggested, which allows evaluating the usefulness of sport in general, or judo, as a means for the development of psychological resilience, as well as the possible benefits of such interventions on sports performance.

## Data availability statement

The raw data supporting the conclusions of this article will be made available by the authors, without undue reservation.

## Ethics statement

The studies involving humans were approved by Ethics Research Committee of Universidad Politécnica de Madrid (Ref. number FDRED00000-DML-DATOS-20230609). The studies were conducted in accordance with the local legislation and institutional requirements. The participants provided their written informed consent to participate in this study.

## Author contributions

MG-M: Data curation, Investigation, Methodology, Project administration, Resources, Supervision, Writing – original draft, Writing – review & editing. CB-G: Data curation, Investigation, Methodology, Project administration, Resources, Writing – original draft, Writing – review & editing. ID-V: Formal analysis, Investigation, Methodology, Validation, Visualization, Writing – original draft, Writing – review & editing. SG-M: Conceptualization, Data curation, Investigation, Methodology, Writing – original draft, Writing – review & editing. JA-S: Conceptualization, Formal analysis, Funding acquisition, Investigation, Methodology, Project administration, Resources, Supervision, Writing – original draft, Writing – review & editing. GR-R: Conceptualization, Data curation, Funding acquisition, Investigation, Methodology, Supervision, Validation, Visualization, Writing – original draft, Writing – review & editing.
